# Comparison of efficacy and safety of three novel hypoglycemic agents in patients with severe diabetic kidney disease: A systematic review and network meta-analysis of randomized controlled trials

**DOI:** 10.3389/fendo.2022.1003263

**Published:** 2022-10-24

**Authors:** Yijun Li, Ying Hu, Xiaoyuan Huyan, Kang Chen, Bing Li, Weijun Gu, Yiming Mu

**Affiliations:** ^1^ Department of Endocrinology, The First Medical Center of Chinese PLA General Hospital, Beijing, China; ^2^ The First Health Care Department, the Second Medical Center & National Clinical Research Center for Geriatric Diseases, Chinese PLA General Hospital, Beijing, China

**Keywords:** glucagon-like peptidyl-1 receptor agonists, dipeptidyl peptidase-4 inhibitors, sodium-glucose cotransporter two inhibitors, severe chronic kidney disease, diabetes mellitus

## Abstract

**Objective:**

To analyze the efficacy and safety of three novel hypoglycemic agents, glucagon-like peptidyl-1 receptor agonists, dipeptidyl peptidase-4 inhibitors (DPP-4i), and sodium-glucose cotransporter two inhibitors (SGLT2i) in type 2 diabetes mellitus (T2DM) patients with severe chronic kidney disease (CKD) (defined in this study as CKD stage 3 B or above, eGFR< 45 mL/min/1.73 m²) based on important RCTs to date.

**Methods:**

We retrieved studies published before April 15, 2022, from EMBASE, PubMed/MEDLINE, Cochrane Library and included randomized controlled trials in which the participants were patients with T2DM and severe CKD. Frequentist methods were used in the network meta-analysis.

**Results:**

Nineteen studies of 17 trials involving 6,607 participants met our inclusion criteria. Compared with placebo and DPP-4i, SGLT2i demonstrated a significantly lower incidence of serious renal-related adverse events or renal death, and the odds ratios (OR) were 0.69 (0.58, 0.81) and 0.63 (0.40, 1.00), respectively. Compared with placebo, SGLT2i significantly reduced the incidence of all-cause death and severe AE; the ORs were 0.72 (0.55, 0.94) and 0.65 (0.47, 0.91), respectively. Compared with placebo, DPP-4i significantly reduced the level of HbA1c, and the difference between mean changes from baseline was -0.36 (-0.63, -0.09).

**Conclusions:**

Patients with T2DM complicated by severe CKD may benefit from SGLT2i. SGLT2i can reduce the incidence of serious renal-related AEs or renal death, as well as severe side effects, and has a positive effect on the patient’s renal function and survival, even for only CKD patients can also be considered. GLP-1 RAs can be used as a supplement if blood sugar control is poor. For dialysis patients, DPP-4i can assist blood glucose control, reduce insulin dosage, and reduce the risk of hypoglycemia.

**Systematic review registration:**

INPLASY https://inplasy.com/inplasy-2021-12-0106/, identifier INPLASY2021120106.

## 1 Introduction

The International Diabetes Federation estimates that 463 million adults aged 20–79 years had diabetes mellitus (DM) globally in 2019, and this number is expected to reach 700 million by 2045. Approximately 90% of patients with DM have type 2 diabetes mellitus (T2DM) (1). Diabetic kidney disease (DKD) accounts for approximately 25% of diabetic cases and is the leading cause of chronic kidney disease (CKD) and end-stage renal disease (ESRD) in most countries. The pathogenesis of DKD is complex, and its clinical manifestations include a continuous increase in urinary albumin excretion and a progressive decrease in estimated glomerular filtration rate (eGFR). Although there is no cure for CKD, its progress can be delayed by controlling risk factors such as hypertension, hyperglycemia, and dyslipidemia (2). T2DM in patients with severe CKD (defined in this study as CKD stage 3 B or above, eGFR< 45 mL/min/1.73 m2) is more challenging to treat since renal damage may affect drug clearance through the kidney, leading to interruption or reduction of most hypoglycemic therapies. In particular, the pharmacological effects of these drugs are difficult to predict in T2DM patients undergoing hemodialysis as the accumulation and rapid clearance of antidiabetic drugs or their metabolites during hemodialysis makes it difficult to maintain blood glucose control (3–5). Therefore, it is particularly difficult to provide a scheme for optimal glycemic control in patients with severe CKD. In addition, the control of blood glucose while protecting renal function and reducing mortality is also worthy of further research. In the past few decades, three novel hypoglycemic agents, glucagon-like peptidyl-1 receptor agonists (GLP-1RAs), dipeptidyl peptidase-4 inhibitors (DPP-4i), and sodium-glucose cotransporter 2 inhibitors (SGLT2i) have been introduced. They have become widely used in the treatment of T2DM. The 2020 Guidelines (6) of KDIGO recommended SGLT2i for patients with eGFR ≥ 30 mL/min/1.73 m2. GLP-1RAs are safe for patients with CKD but are not recommended for ESRD patients. DPP-4i reduces blood glucose and lowers the risk of hypoglycemia, but there is no evidence that it improves renal or cardiovascular outcomes. After completing several large RCTs, some new findings have been made regarding the efficacy of the three novel hypoglycemics in patients with severe DKD and ESRD. Research by Heerspink et al. (7) revealed that dapagliflozin significantly reduced the incidence of death from renal or cardiovascular causes in DKD patients with eGFR<45 mL/min/1.73 m2 than that in patients who were administered a placebo. Another study showed that dialysis patients taking saxagliptin had significantly lower HbA1c levels than controls (8). However, there is insufficient evidence to conclude whether these agents are effective for T2DM patients with severe CKD.

The purpose of this study was to analyze the efficacy and safety of the three novel hypoglycemics in T2DM patients with severe CKD, based on important RCTs to date. Due to the lack of head-to-head trials, this study used network meta-analysis.

## 2 Methods

We followed the PRISMA guidelines for network meta-analysis (9). The study protocol was published in the INPLASY database under the registration number INPLASY2021120106 (https://inplasy.com/inplasy-2021-12-0106/).

### 2.1 Eligibility criteria

We included RCTs with parallel-group design. The participants were T2DM patients with severe CKD. If the study population in the subgroup met the above criteria, the study was considered eligible. Studies included at least one of the following treatments: GLP-1RA, DPP-4i, and SGLT2i. The results of eligible studies included changes in HbA1c levels from baseline, hypoglycemic events, renal-related adverse events (AEs) (consisting of renal replacement therapy, acute kidney injury, and acute kidney failure), or death. We excluded single-arm studies and positive control studies for the two drugs in the same category.

### 2.2 Identification of studies

We searched for articles published before April 15, 2022. Electronic searches were conducted in Embase, PubMed/Medline, and the Cochrane Library. The search terms used are presented in the Supplementary Method S1 section of the supplementary materials. The two authors conducted an independent literature search, screened titles and abstracts, and read the full text to determine whether the study met the inclusion criteria. The PRISMA flow diagram was used to summarize the study selection process.

### 2.3 Data extraction and efficacy measures

One author extracted relevant information from qualified trials, and the other author independently reviewed the data. The differences were resolved by consensus through discussion. The extraction included author, year of publication, country, stage of CKD, age, sex percentage, drug use, sample size, and follow-up. For data that were not available in digital form, we used the free software PlotDigitizer (https://plotdigitizer.com/app)to extract digital data from graphics. In some cases, we obtained standard deviations from standard errors or 95% CIs and classified data from individual patient data or percentages. We also obtained the required data from publications by different authors of the same trial. The primary outcome was the incidence of serious renal-related AEs or renal death, other outcomes were assessed as serious AEs, hypoglycemia, severe hypoglycemia, all-cause mortality, and changes in HbA1c compared with baseline. We performed a subgroup analysis of patients with ESRD undergoing hemodialysis.

### 2.4 Statistical analysis

This network meta-analysis was performed using a frequentist random-effects model (10). We ranked medications according to the surface under the cumulative ranking (SUCRA) (11). A higher SUCRA value indicated better performance. Odds ratio (OR) was used to pool effect sizes for adverse events, weighted mean difference was used to pool effect sizes for HbA1c change, and interval estimation was performed using 95% CIs. The level of significance was set at an α value of 0.05. We assessed the transitivity assumption by analyzing the distribution or frequency of potential effect modifiers in treatment: HbA1c at baseline, age, and proportion of men. We conducted random effects (DerSimonian- Laird estimator) pairwise meta-analyses for all comparisons, allowing for heterogeneity in effect size between studies. The proportion of the total variance within each pairwise comparison that is due to between study heterogeneity was estimated using the I² statistic. We evaluated the small-study effects by visually observing publication bias using a comparison-adjusted funnel plot (12). The mvmeta command and network packages of commands in Stata software were used for this analysis (13, 14). Pairwise meta-analysis with a random-effects model was used to perform subgroup analysis. All analyses were performed using Stata version 15.0.

### 2.5 Assessment of risk of bias in individual studies

The quality of the retrieved RCTs was assessed according to the Cochrane Handbook of Systematic Reviews of Interventions (15). Potential sources of bias include sequence generation (selection bias), allocation sequence concealment (selection bias), blinding of participants and personnel (performance bias), blinding of outcome assessment (detection bias), incomplete outcome data (attrition bias), and selective outcome reporting (reporting bias). The two authors independently conducted this assessment, and studies were graded as high risk, low risk, or uncertain risk.

## 3 Results

### 3.1 Study selection

Electronic retrieval of the database yielded 5,024 studies. After reading the full text, 19 studies on 17 trials covering 6607 participants met our inclusion criteria (EMPA-REG OUTCOME trial was included in two studies and the DAPA-CKD trial in two studies) (in **Supplementary Figure 1**).

### 3.2 Study characteristics and risk of bias within studies

In the 17 RCTs, SGLT2i, DPP-4i, and GLP-1RA were used as pharmacological interventions in five, nine, and three trials, respectively. The average age of the patients was 60-70 years. The baseline of patients adopted in the subgroup of this study was eGFR < 45 mL/min per 1.73m². Detailed information on each study is listed in **Table 1**. **Supplementary Figures 2**-**8** show the network for all the included trials.

**Table 1:** Characteristics of included studies.

Study	Year	Age	Male%	eGFR(ml/min/1.73m2)	Medicines	Sample size	Country	Follow up(week)	Registration	HbA1c	Background antidiabetes
Abe (8)	2016	66.9 (9.4)	67.1%	eGFR < 15 undergoing hemodialysis	Saxagliptin	82	Japan	24	UMIN000018445	6.5 ± 0.8	conventional antidiabetic drugs (oral drugs and/or insulin)
Arjona Ferreira (16)	2013	60.5 (9.1)	59.7%	< 15	Sitagliptin	129	USA	54	NCT00509236	7.9 ± 0.7	glipizide
Barnett (17)	2014	62·9 (11·9)	51.4%	15-30	Empagliflozin	74	UK	52	NCT01164501	8.0 ± 0.8	conventional antidiabetic drugs (oral drugs and/or insulin)
Chacra (18)	2017	65.9(9.4)	63.6%	eGFR 15- 30 not undergoing hemodialysis +eGFR < 15 undergoing hemodialysis	Omarigliptin	98	USA	24+30	MK-3102-019, NCT01698775	8.3 ± 0.8	None
Davies (19)	2016	68.0 (8.3)	53.6%	30-45	Liraglutide	120	UK	26	LIRA-RENAL NCT01620489	8.1 ± 0.8	conventional antidiabetic drugs (oral drugs and/or insulin)
Heerspink (7)	2020	61.8(12.1)	67.1%	25-45	Dapagliflozin	2522	Netherlands	2.4 years	DAPA-CKD NCT03036150	7.1 ± 1.7	None
Heerspink (20)	2021	61.8(12.1)	67.1%	25-45	Dapagliflozin	2522	Netherlands	2.4 years	DAPA-CKD NCT03036150	7.1 ± 1.7	None
Herrington (21)	2018	67.1(7.6)	67.3%	20-45	Empagliflozin	570	UK	3.1 years	EMPA-REG OUTCOME	8.1 ± 0.9	conventional antidiabetic drugs (oral drugs and/or insulin)
Idorn (22)	2015	68.3(3.1)	80.0%	< 15	Liraglutide	20	Denmark	12	NCT01394341	6.7 ± 0.4	insulin dose was reduced by 20–50% at sulphonylureas were paused, while metformin was continued in unchanged doses
Ito (23)	2011	67 (2)	70.0%	eGFR < 15 undergoing hemodialysis	Vildagliptin	51	Japan	24	–	6.7 ± 0.5	conventional antidiabetic drugs (no insulin)
Kothny (24)	2012	63.7(9.1)	52.1%	eGFR<30, including undergoing hemodialysis	Vildagliptin	146	Finland	52	–	7.9 ± 1.0	untreated, insulin, oral antidiabetic drugs or any combination
Mann (25)	2017	64.2	64.5%	15- 30	Liraglutide	224	Germany	3.84 years	NCT01179048	8.7 ± 1.5	glucose-lowering therapies excluding the use of DPP-4i or GLP-1 analogs
McGill (5)	2013	64.0(10.9)	66.2%	<30	Linagliptin	133	USA	1 year	NCT00800683	8.2 ± 1.1	untreated, insulin, oral antidiabetic drugs or any combination
Munch (26)	2020	70.5 (8.5)	52.3%	eGFR < 15 undergoing hemodialysis	Vildagliptin	65	France	12	VILDDIAL NCT02176681	7.3 ± 1.1	insulin
Neuen (27)	2018	68.7 (8.0)	55.8%	30 - 45	Canagliflozin	554	Australia	188	CANVAS NCT01032629, NCT01989754	8.3 ± 1.0	glucose-lowering therapies excluding the use of SGLT2i
Nowicki (28)	2011	66.8 (8.3)	37.6%	CrCl <30 ml/min +eGFR < 15	Saxagliptin	170	Poland	12	NCT00614939	8.5 (1.2)	Oral antihyperglycaemic drugs and insulin therapy
Perkovic (29)	2019	63.0(9.2)	66.1%	30 - 45	Canagliflozin	1313	Australia	2.62 years	CREDENCE NCT02065791	8.3 ± 1.3	None
Udell (30)	2015	70 (64–75)	55.8%	< 30	Saxagliptin	336	Canada	2 years	SAVOR-TIMI 53 NCT01107886	7.6 (1.0)	glucose-lowering therapies excluding the use of DPP-4i or GLP-1 analogs
Wanner (31)	2018	67.1(7.6)	67.3%	20 - 45	Empagliflozin	570	Germany	3.1 years	EMPA-REG OUTCOME NCT01131676	8.1 ± 0.9	conventional antidiabetic drugs (oral drugs and/or insulin)
Total studies	19					6607					

DPP-4i, dipeptidyl peptidase-4 inhibitors; eGFR, estimated glomerular filtration rate; GLP-1, glucagon-like peptidyl-1; SGLT2i, Sodium-glucose cotransporter 2 inhibitors.

Among the 17 RCTs, two were open-label and had a high risk of performance bias. Two trials had a high risk of attrition bias. One trial had a high risk of selection bias because of the imbalance between the intervention and control groups in the severe CKD subgroup. Most studies had a low risk of bias. Details of the quality assessment are illustrated in **Supplementary Figures 9**, **10**.

### 3.3 Homogeneity and transitivity assumption

The mean age of the included studies was between 60 and 70 years old, with the maximum being 70.5 ± 8.5 and the minimum being 60.5 ± 9.1; The proportion of men is more than that of women, between 50% and 80%, while in one study, the proportion of men is 37%. **Supplementary Figures 11**, **12**. In addition, the mean level distribution of baseline HbA1c is 6.5 ± 0.8 to 8.7 ± 1.5, therefore the baseline HbA1c of subjects is very similar, **Supplementary Figure 13**. The intervention measures of the study were similar: comparing the studied agents with placebo on the basis of background hypoglycemic therapy, and there were 2 trails without any background hypoglycemic, **Table 1**. The quality of the research was also balanced, as shown in **Table 1**. As such, the assumption of transitivity is likely to hold in our data.

We found some evidence of statistical heterogeneity (I² statistic > 50%) within pairwise comparisons, however, only one I² statistic was greater than 85%. We believe that the homogeneity assumption of both pairwise and network meta-analysis is met, **Supplementary Table 1**.

### 3.4 Synthesis of results

This study compared the incidence of various serious AEs and evaluated the safety of the three novel hypoglycemics GLP-1RA, DPP-4i, and SGLT2i in T2DM patients with severe CKD. The changes in HbA1c from baseline were also compared to observe the efficacy of these three novel hypoglycemics in reducing blood glucose.

### 3.5 Pairwise meta-analyses

**Supplementary Table 1** presents the results of the pairwise meta-analysis and heterogeneity estimates. Briefly, Compared with placebo, SGLT2i demonstrated a significantly lower incidence of renal-related adverse events and all-cause mortality, ORs were 0.686(0.581, 0.810) and 0.719(0.548, 0.944), respectively. Compared with placebo, DPP-4i and GLP1 demonstrated significant reduction in HbA1c, with mean difference of -0.362(-0.634, -0.089) and -0.491(-0.835, -0.147), respectively.

### 3.6 Network meta-analysis

#### 3.6.1 Serious renal-related adverse events or renal death

**Figure 1:**
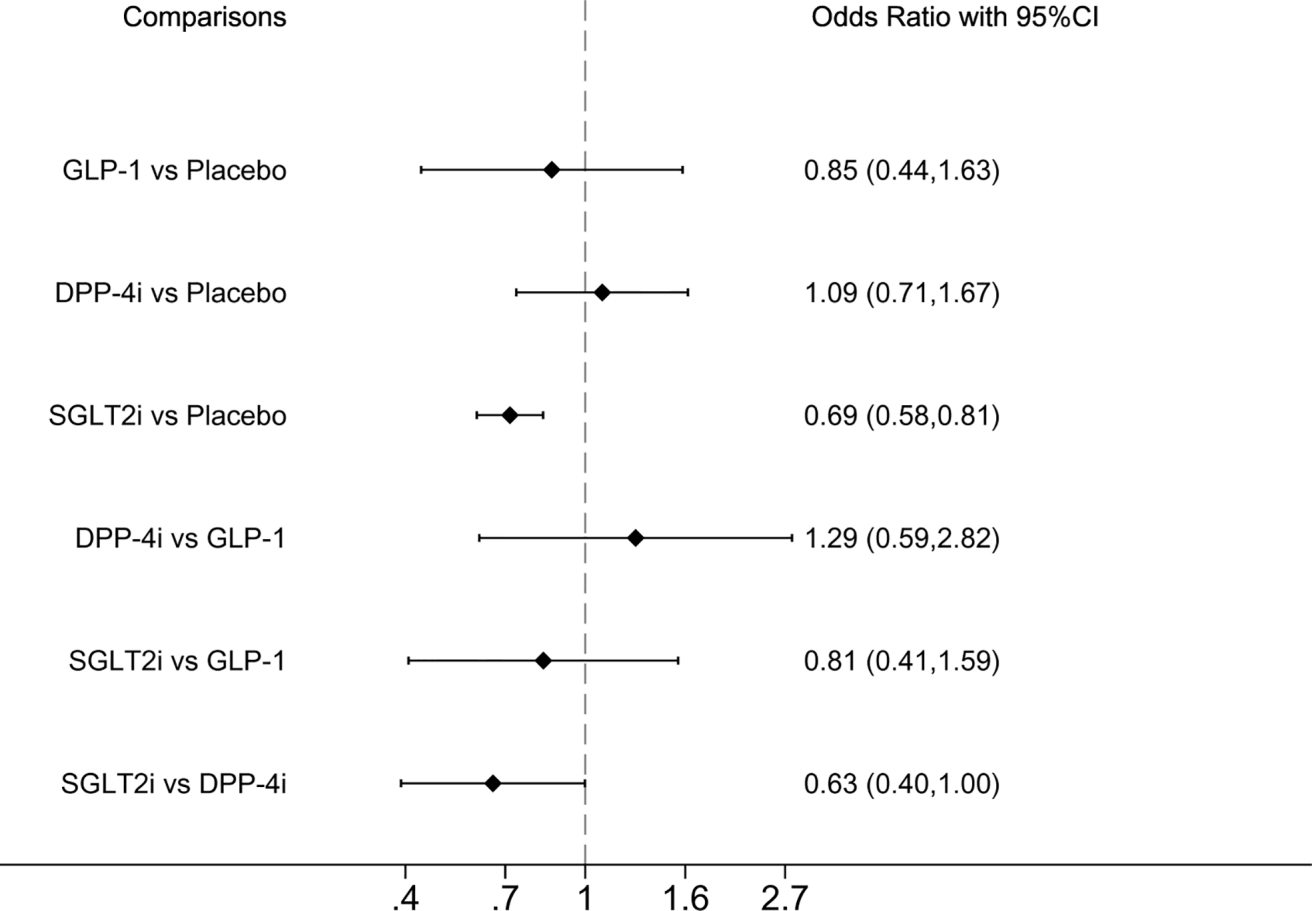
Forest plot of odds ratio comparing the serious renal-related adverse events or renal death between each medicine class or between each medicine class and placebo.

Serious renal-related AEs or renal deaths were reported in seven studies. Compared with placebo and DPP-4i, SGLT2i demonstrated significantly lower incidence of serious renal-related AEs or renal death, ORs were 0.69 (0.58, 0.81) and 0.63 (0.40, 1.00), respectively (**Figure 1**). SUCRA ranking showed that SGLT2i performed best and placebo performed the worst, as shown in **Table 2**.

**Table 2:** SUCRA.

Treatment	SUCRA	PrBest	MeanRank
**Serious renal-related adverse events or renal death**			
Placebo	32.6	0	3
GLP-1	56.7	26.5	2.3
DPP-4i	20.4	1.9	3.4
***SGLT2i***	***90.3***	***71.6***	***1.3***
**Serious AE**			
Placebo	39.4	0.3	2.8
GLP-1	21.8	7.7	3.3
DPP-4i	43.9	6.1	2.7
***SGLT2i***	***94.9***	***85.9***	***1.2***
**Hypoglycemia**			
***Placebo***	***73.2***	***36.9***	***1.8***
GLP-1	7.7	5.1	3.8
DPP-4i	64.8	33	2.1
SGLT2i	54.3	25	2.4
**Severe hypoglycemia**			
Placebo	32.7	10.7	2.3
DPP-4i	52.5	37.6	2
***SGLT2i***	***64.9***	***51.7***	***1.7***
**All-cause mortality**			
Placebo	17.6	0.4	2.6
DPP-4i	51.6	37.4	2
***SGLT2i***	***80.8***	***62.2***	***1.4***
**Change from baseline in HbA1c, %**			
Placebo	23.9	0.1	3.3
GLP-1	74.1	50.6	1.8
***DPP-4i***	***79.8***	***45.2***	***1.6***
SGLT2i	22.3	4.1	3.3

The bold italics indicate the best performance. AE, Adverse event; DPP-4i, dipeptidyl peptidase-4 inhibitors; GLP-1, glucagon-like peptidyl-1; SGLT2i, Sodium-glucose cotransporter 2 inhibitors; SUCRA, Surface under the cumulative ranking.

#### 3.6.2 Serious AE

**Figure 2:**
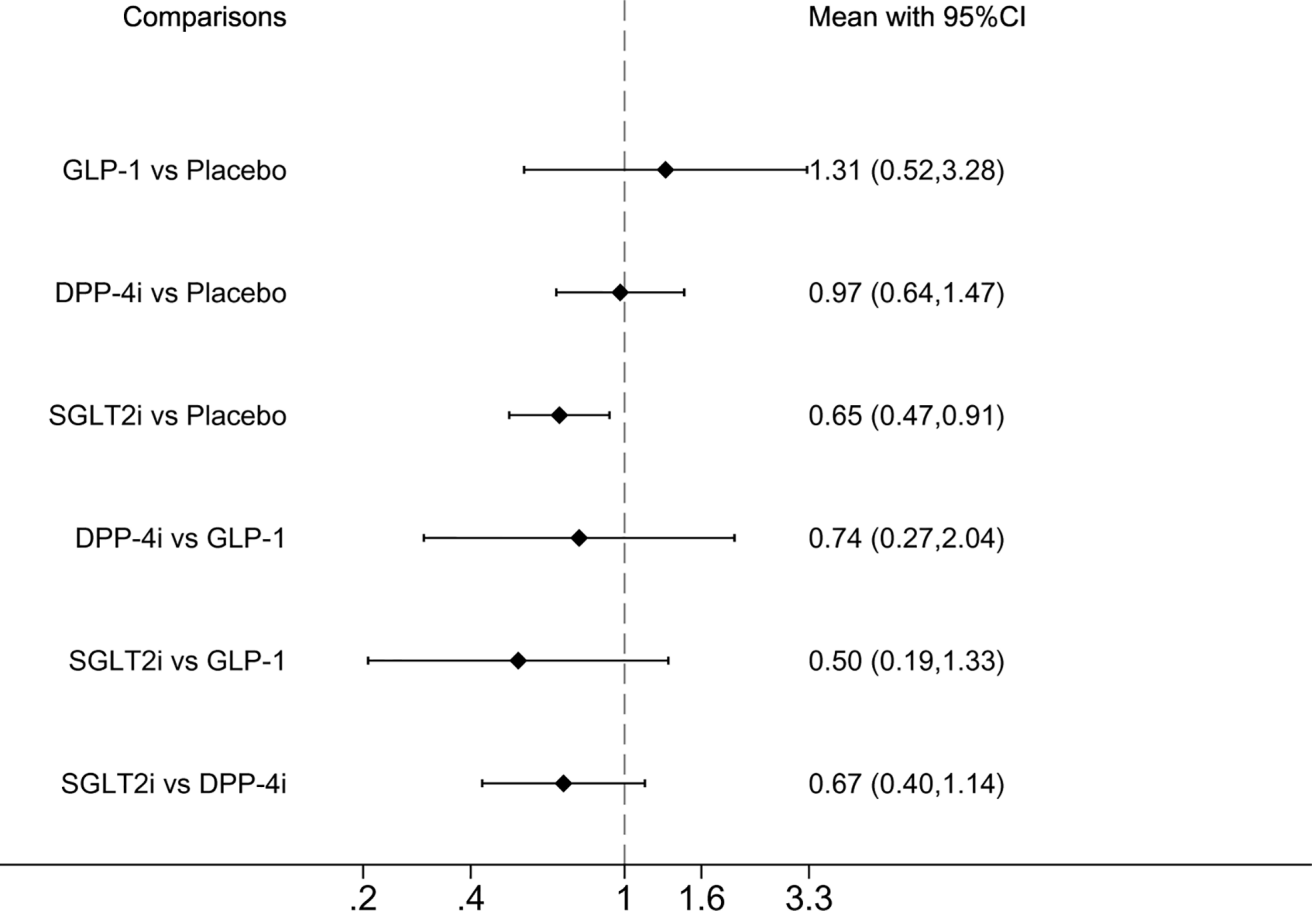
Forest plot of odds ratio comparing the serious adverse events between each medicine class or between each medicine class and placebo.

Serious AEs (defined as medical events that resulted in death, hospitalization, or significant disability or incapacity, and were identified by searching in the Medical Dictionary for Regulatory Activities) (23, 31) were reported in eight studies. Compared with placebo, SGLT2i demonstrated a significantly lower incidence of serious AEs OR was 0.65 (0.47, 0.91) ((**Figure 2**). SUCRA ranking showed that SGLT2i performed best and GLP-1 performed the worst (**Table 2**).

#### 3.6.3 All-cause mortality

**Figure 3:**
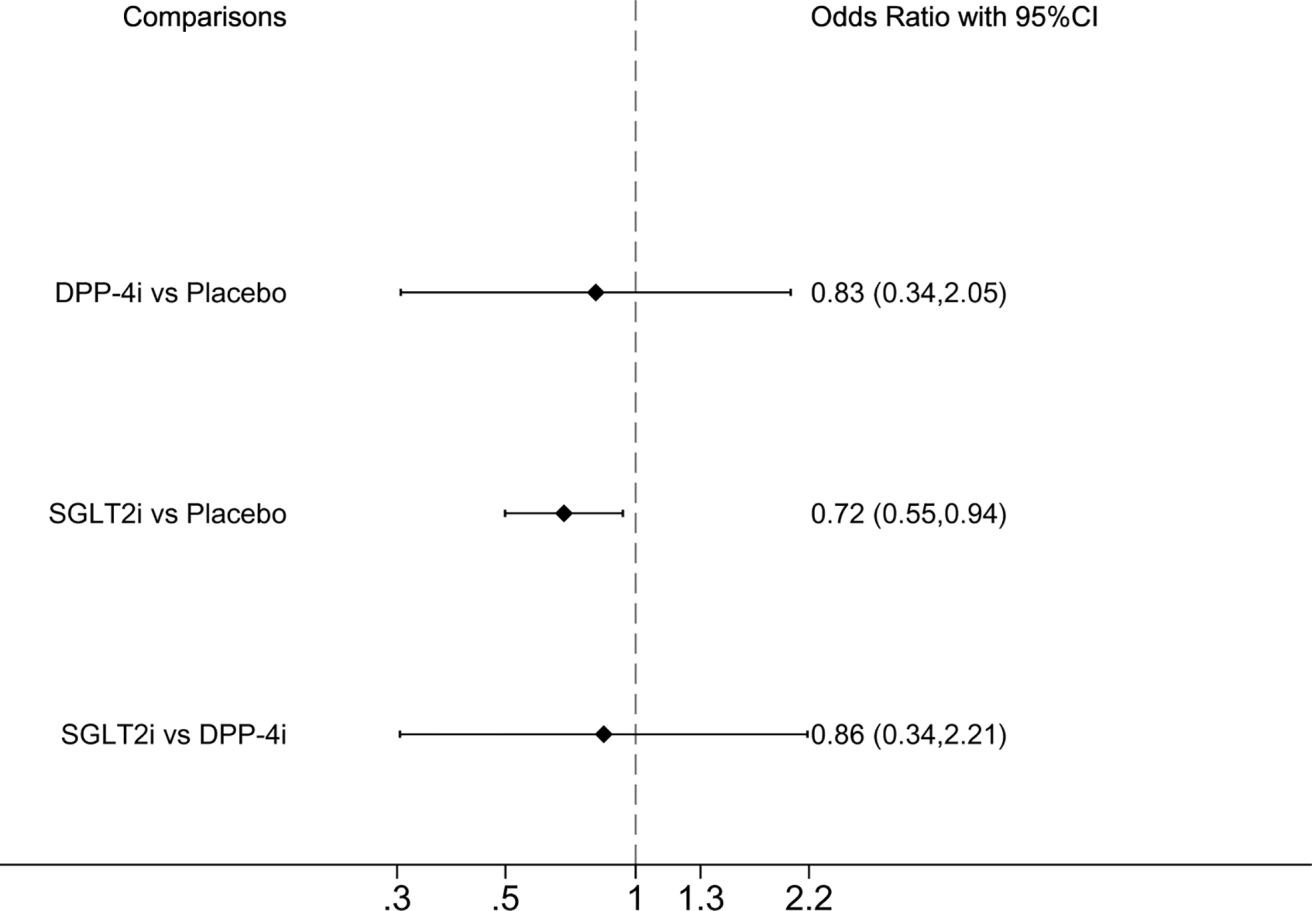
Forest plot of odds ratio comparing the all-cause mortality between each medicine class or between each medicine class and placebo.

All-cause mortality was reported in seven studies, whereas GLP-1RA was absent. Compared with placebo, SGLT2i demonstrated a significantly lower incidence of all-cause mortality, OR of 0.72 (0.55, 0.94] ((**Figure 3**). SUCRA ranking showed that SGLT2i performed best and placebo performed the worst (**Table 2**).

#### 3.6.4 Hypoglycemia and severe hypoglycemia

There were no significant differences between the three novel hypoglycemic and placebo groups in the incidence of hypoglycemia or severe hypoglycemia, nor were there significant differences between the three novel hypoglycemics themselves (in **Supplementary Figures 14**, **15**). This suggests that these three drugs are equally likely to cause adverse events of hypoglycemia in T2DM patients with severe CKD.

#### 3.6.5 HbA1c

The mean changes from baseline HbA1c levels were reported in 12 studies. Compared with placebo, DPP-4i demonstrated a significant reduction in HbA1c, with a mean difference of 0.36 (-0.63, -0.09) (in **Supplementary Figure 16**). The SUCRA ranking showed that DPP-4i performed best and placebo performed the worst (**Table 2**).

#### 3.6.6 Subgroup analysis

Due to the lack of data on the efficacy and safety of SGLT2i and GLP-1RA in dialysis patients, we only performed subgroup analyses of DPP-4i in this population. Six studies reported mean changes from baseline HbA1c levels. Compared with placebo, DPP-4i demonstrated a significant reduction in HbA1c in patients undergoing dialysis, MD was -0.22 (-0.42, -0.03) (in **Supplementary Figure 17**). Three studies reported the incidence of hypoglycemia in dialysis patients treated with DPP-4i versus placebo, with no difference in the pooled results, OR was 0.88 (0.34, 2.29), in **Supplementary Figure 18**.

#### 3.6.7 Publication bias assessment

No publication bias was found by visual inspection of the funnel plots (in **Supplementary Figure 19**).

## 4 Discussion

### 4.1 SGLT2i is feasible in CKD patients with stage 4 disease

In this study, compared with placebo, SGLT-2i demonstrated significantly lower all-cause mortality, the incidence of serious renal-related AEs or renal death, and severe side effects. Although there was no significant difference between the three drugs, SGLT2i had the highest SUCRA ranking and the best efficacy. SGLT2i is a novel oral antidiabetic drug that is distinguished from other traditional hypoglycemics. SGLT2i can act on renal tubules and exert glucose-lowering effects by inhibiting renal tubular reabsorption of glucose and promoting urinary glucose excretion. Theoretically, the hypoglycemic effect of SGLT2i usually declines as kidney function declines (32). Thus, many physicians are skeptical about the use of SGLT2i in patients with stage 4 disease.

Nevertheless, patients with stage 4 disease were covered by all three included studies (7, 17, 21), and our findings illustrated that even CKD patients with stage 4 can still benefit from SGLT2i. However, the SUCRA ranking in this study showed that SGLT2i had the worst performance in reducing HbA1c, all-cause death, and severe kidney injury were still significantly lower. This illustrates from another perspective that the renal protection of SGLT2i is not entirely dependent on blood glucose control but is most likely through direct renal mechanisms to benefit the kidneys. Reliable evidence is still lacking regarding the renal protective effect of SGLT2i, but several hypotheses have been proposed to explain the observed phenomenon. One of the proposed mechanisms is an improved glomerular hemodynamics due to increased adenosine levels caused by an increase in membrane Na+/K+ ATPase activity, which leads to afferent arteriole constriction, thereby reducing glomerular hyperfiltration, reducing proteinuria, and preserving renal function in the long term (33–36). SGLT2i may also protect the kidneys in other ways, including enhancing oxygenation of the kidney by reducing tubular energy requirements, metabolic and anti-inflammatory effects, and directly affecting glomerular endothelial function (37, 38). Therefore, we believe that SGLT2i is not completely dependent on the function of blood sugar control and is still capable of renal protection, making it feasible to use in CKD stage 4 with T2DM patients; moreover, it can have a positive effect on the patient’s renal function and survival, even for only CKD patients can also be considered.

### 4.2 GLP-1 RAs can be administered as a supplement to achieve glycemic control in patients with severe CKD

GLP-1 RAs (including exenatide, liraglutide, and lixisenatide) exert hypoglycemic effects by enhancing insulin secretion and inhibiting glucagon secretion through the activation of GLP-1 receptors. Lixisenatide and exenatide are eliminated mainly through glomerular filtration and subsequent proteolytic degradation in the renal tubules (39–42). However, they are not suitable for patients with advanced renal insufficiency. However, liraglutide is so unique that it binds plasma proteins extensively (98%) and is metabolized similarly to large proteins, with no specific organ identified as the main elimination site. The kidneys excrete only a small portion (6%). Liraglutide was covered by all the GLP-1RA studies we included (19, 22, 25). In our study, GLP-1 RAs did not differ from placebo concerning serious renal-related AEs or renal death in patients with severe CKD. GLP-1 RAs ranked better than DPP-4i and placebo, but lower than placebo SGLT2i in SUCRA rankings. In the SUCRA ranking, the effect of GLP-1 RAs in reducing HbA1c was second only to DPP-4i and better than SGLT2i and placebo. Notably, Idorn et al. (22) found that using GLP-1 RAs in patients with ESRD significantly reduced the basal insulin dose during treatment without worsening glycemic control. This suggests that GLP-1 RAs may be suitable for patients with ESRD, although larger studies are needed to confirm this finding. The 2020 KDIGO guidelines (6) recommend using long-acting GLP-1RA for T2DM patients with CKD who have not achieved individualized glycemic targets with metformin and SGLT2i or are unable to take these agents. We believe that GLP-1 RAs can be added to maintain blood glucose levels when SGLT2i cannot achieve the blood glucose targets in stage 3 B and stage 4 CKD patients. It not only benefits from the reno-protective effects of SGLT2i but also ensures the control of blood glucose by GLP-1 RAs.

### 4.3 DPP-4i can still play a hypoglycemic effect in patients undergoing dialysis

In the subgroup analysis of this study, it was found that DPP-4i significantly reduced HbA1c and did not increase the risk of hypoglycemia in dialysis patients compared with placebo. The mechanism of DPP-4i includes stimulation of insulin and glucagon, indicating that its combination with other hypoglycemics can further reduce HbA1c. Some researchers have recently raised doubts about HbA1c as a blood glucose indicator. HbA1c is a long-term biomarker that reflects blood glucose levels over the lifespan of red blood cells. Notably, CKD is associated with diseases such as oxidative stress, metabolic acidosis, and inflammation, which, in addition to hyperglycemia, may simultaneously promote the formation of advanced glycation end products, leading to elevated HbA1c levels (43).

On the contrary, due to erythropoiesis stimulants or iron replacement therapy, blood transfusion, and anemia, the survival or age of erythrocytes is shortened, and HbA1c is lowered (43, 44). These effects were most significant in patients with advanced CKD, especially those undergoing dialysis. Despite some limitations, no better biomarkers have been found to replace HbA1c. Therefore, the 2020 KDIGO guidelines (6) recommend that HbA1c blood glucose monitoring is appropriate for all adults, children, and patients with renal failure treated by dialysis or renal transplantation. Therefore, when evaluating the hypoglycemic effect by reducing HbA1c in patients, we believe that DPP-4I is better than the other two drugs and is equally effective in dialysis patients.

### 4.4 Strengths and limitations

To our knowledge, this is the first network meta-analysis of the efficacy and safety of three novel hypoglycemics in T2DM patients with severe CKD. Previous meta-analyses have compared the benefits of SGLT2i and GLP-1RAs in patients with CKD (eGFR < 60 ml/min/1.73 m2) (45). Two meta-analyses have assessed the efficacy and safety of only DPP-4i in T2DM patients with moderate to severe renal impairment (46, 47). This meta-analysis provides evidence for this under-lighted area. Nevertheless, this study had several limitations. First, the patients in this study were all from subgroups of RCTs included; patients with severe CKD might be less randomized. Second, only DPP-4i was evaluated in the subgroup analysis of dialysis patients because of little data, but the efficacy of GLP-1 and SGLT2i in dialysis patients was not evaluated. Third, since the included RCTs did not compare the three drugs head-to-head, this network meta-analysis did not test the inconsistency by comparing the results of indirect and direct comparisons. Therefore, the findings of this study should be interpreted with caution. Perhaps what we can expect is more head-to-head clinical trials in the future.

## 5 Conclusions

In conclusion, we believe that T2DM patients with severe CKD may benefit from SGLT2i. SGLT2i can reduce the incidence of serious renal-related AEs or renal death, as well as severe side effects, and has a positive effect on the patient’s renal function and survival, even for only CKD patients can also be considered. If blood glucose control is poor, GLP-1 RAs can be administered as a supplement, but this is not recommended for patients with end-stage renal disease because there is insufficient evidence to support its safety and efficacy in this population. In dialysis patients, DPP-4i can assist blood glucose control, reduce insulin dosage, and reduce the risk of hypoglycemia.

## Data availability statement

The original contributions presented in the study are included in the article/**Supplementary Material**. Further inquiries can be directed to the corresponding authors.

## Author contributions

YL, YH and XH contributed equally to this work. YL, YH, XH, WG and YM conceived and designed the study. KC and BL screened and extracted data. YL, YH and XH performed the statistical analyses. All of the authors contributed to the interpretation of data. YL, YH and XH drafted the manuscript. All of the authors revised it critically for important intellectual content. All authors contributed to the article and approved the submitted version.

## Conflict of interest

The authors declare that the research was conducted in the absence of any commercial or financial relationships that could be construed as a potential conflict of interest.

## Publisher’s note

All claims expressed in this article are solely those of the authors and do not necessarily represent those of their affiliated organizations, or those of the publisher, the editors and the reviewers. Any product that may be evaluated in this article, or claim that may be made by its manufacturer, is not guaranteed or endorsed by the publisher.
