# Supplementary material for: Comparison of efficacy and safety of three novel hypoglycemic agents in patients with severe diabetic kidney disease: A systematic review and network meta-analysis of randomized controlled trials
Source: Front Endocrinol (Lausanne). 2022 Oct 24;13:1003263. doi: 10.3389/fendo.2022.1003263 (PMC9637657; doi:10.3389/fendo.2022.1003263)
Supplement: Supplementary file 1 [file DataSheet_1.pdf]

Supplementary Material

1 Supplementary Data

Method S1: Search terms

(diabetes OR diabetes mellitus OR diabetic*) AND (type 2 OR type II OR type ii OR NIDDM OR noninsulin dependent OR noninsulin dependent OR adult onset OR mature onset OR late onset) OR (diabetic nephropath* OR diabetic kidney disease OR renal) AND (glucagon-like peptide 1 receptor inhibitor* OR glucagon-like peptide 1 receptor agonist* OR glucagon-like peptide 1 inhibitor* OR glucagon-like peptide 1 agonist* OR GLP-1 receptor inhibitor* OR GLP-1 receptor agonist* OR GLP-1 inhibitor* OR GLP-1 agonist* OR dulaglutide OR exenatide OR exendin 4 OR liraglutide OR semaglutide OR taspoglutide OR lixisenatide OR albiglutide OR sodium glucose transporter 2 inhibitor* OR sodium glucose transporter ii inhibitor* OR SGLT 2 inhibitor* OR canagliflozin OR dapagliflozin OR empagliflozin OR ertugliflozin OR tofogliflozin OR bexagliflozin OR henagliflozin OR ipragliflozin licogliflozin OR luseogliflozin OR remogliflozin OR sergliflozin OR sotagliflozin OR dipeptidyl-peptidase IV Inhibitor* OR dipeptidyl-peptidase 4 Inhibitor* OR DPP4 inhibitor* OR DPP 4 inhibitor* OR DPP IV inhibitor* OR omarigliptin OR vildagliptin OR sitagliptin OR saxagliptin OR linagliptin OR alogliptin OR septagliptin OR gemigliptin OR anagliptin OR teneligliptin OR evogliptin OR dutogliptin OR retagliptin) Filters: Clinical Trial, Randomized Controlled Trial

2 Supplementary Figures and Tables

2.1 Supplementary Figures

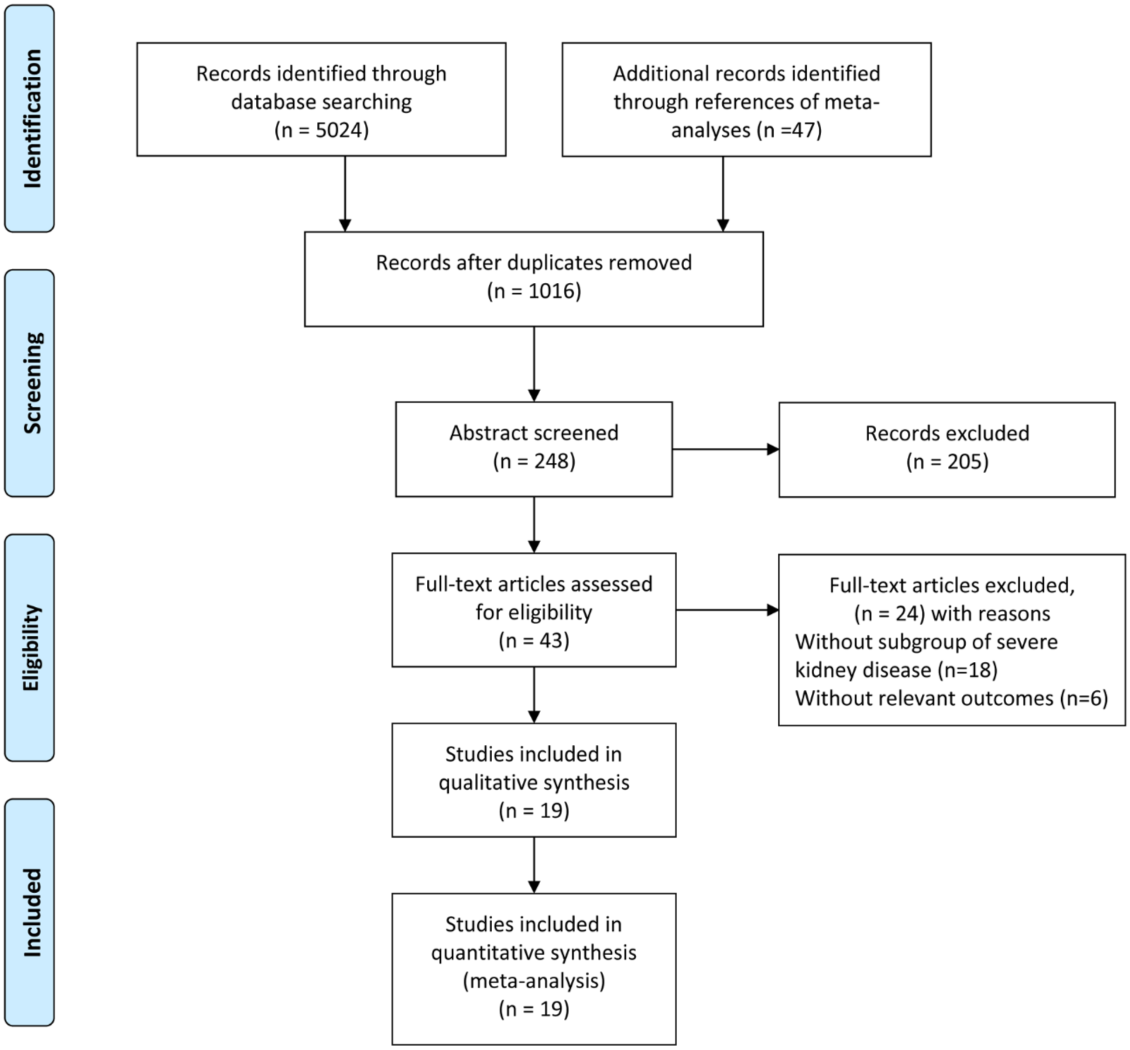

Supplementary Figure 1. Flowchart of study selection

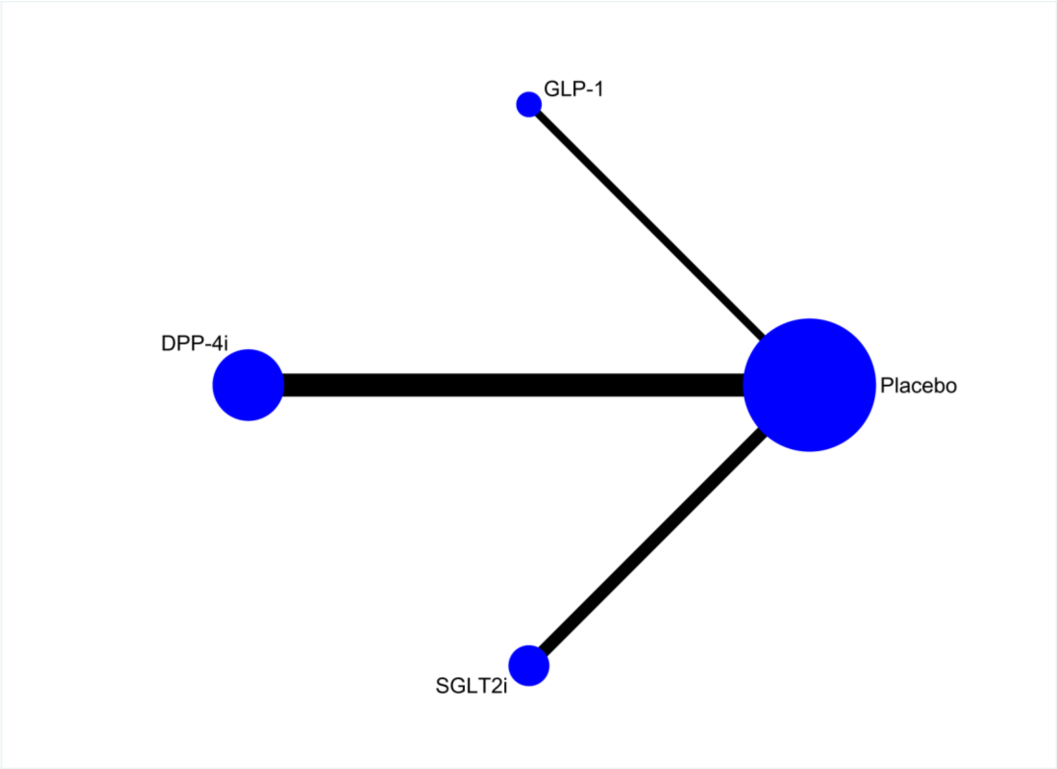

Supplementary Figure 2. Network Plot for all Studies

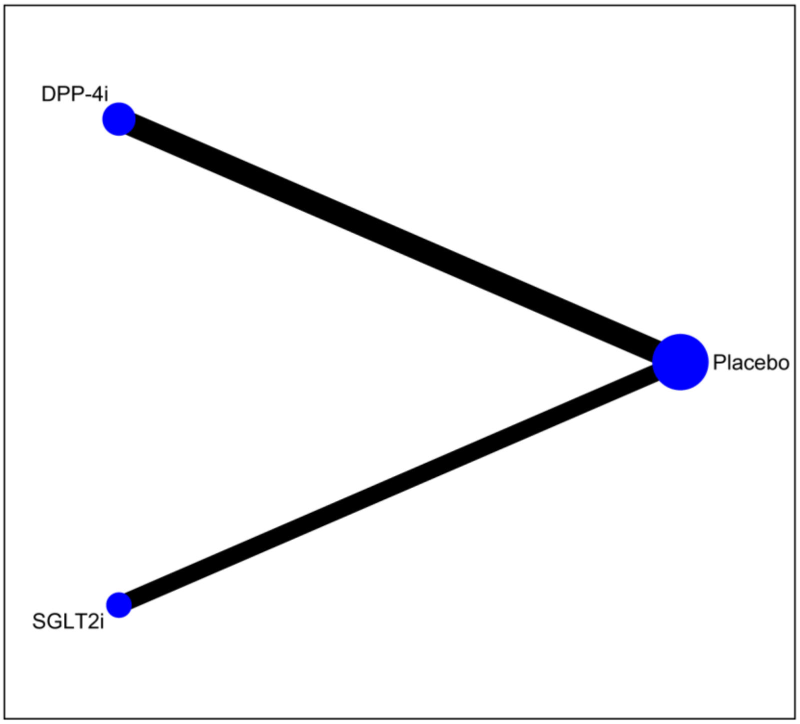

Supplementary Figure 3. Network Plot for NMA of all-cause mortality

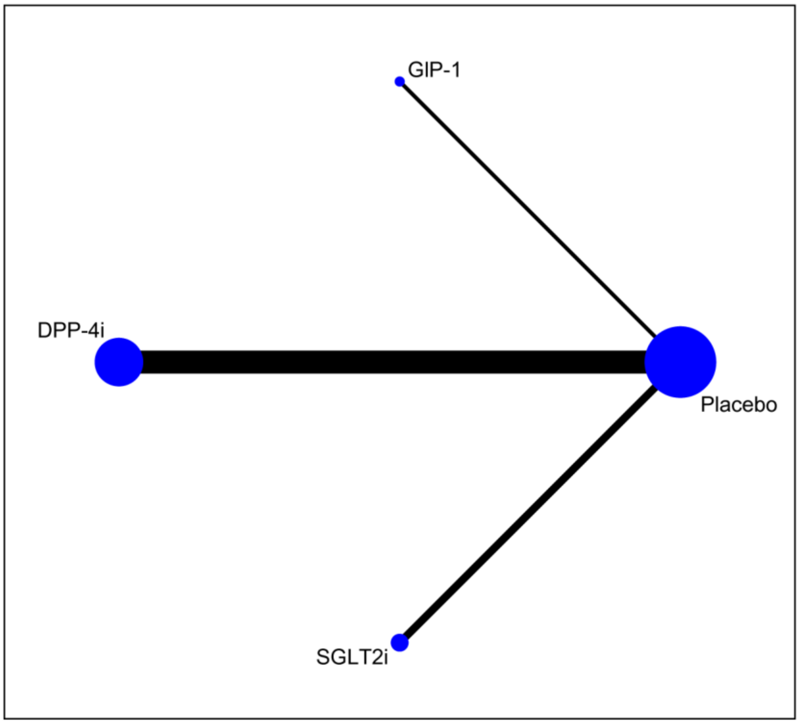

Supplementary Figure 4. Network Plot for NMA of hypoglycemia

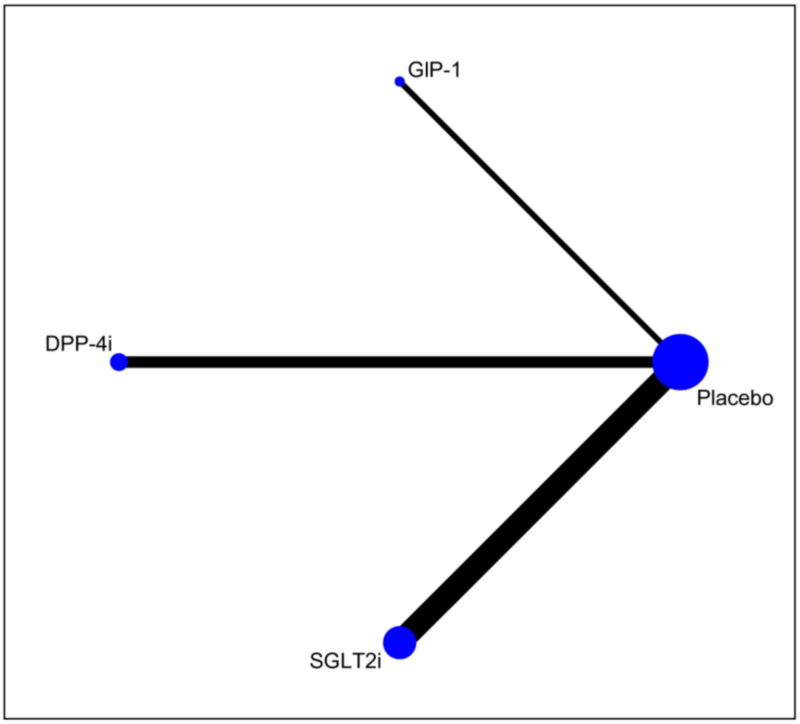

Supplementary Figure 5. Network Plot for NMA of serious renal-related adverse events or renal death

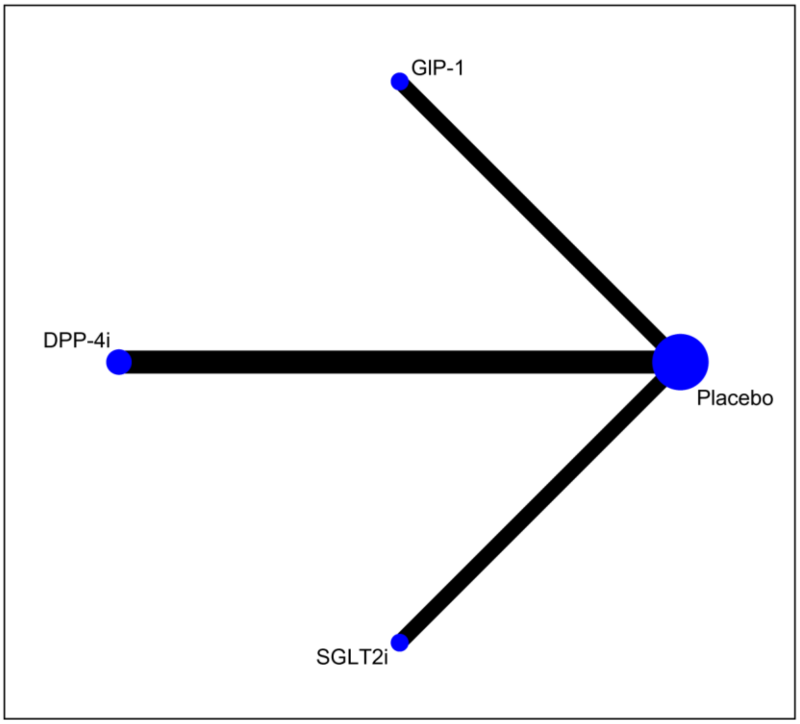

Supplementary Figure 6. Network Plot for NMA of serious AE

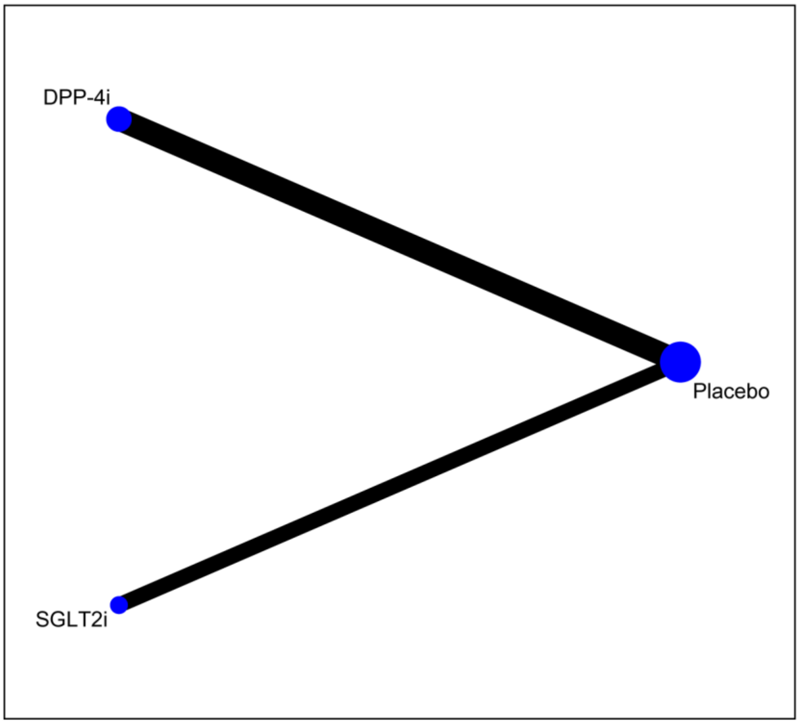

Supplementary Figure 7. Network Plot for NMA of severe hypoglycemia

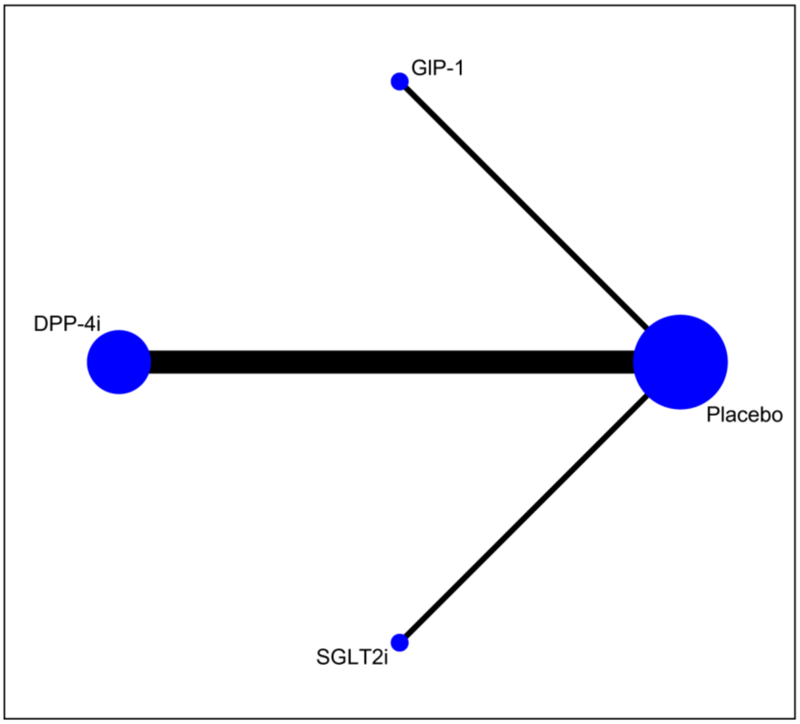

Supplementary Figure 8. Network Plot for NMA of HbA1c

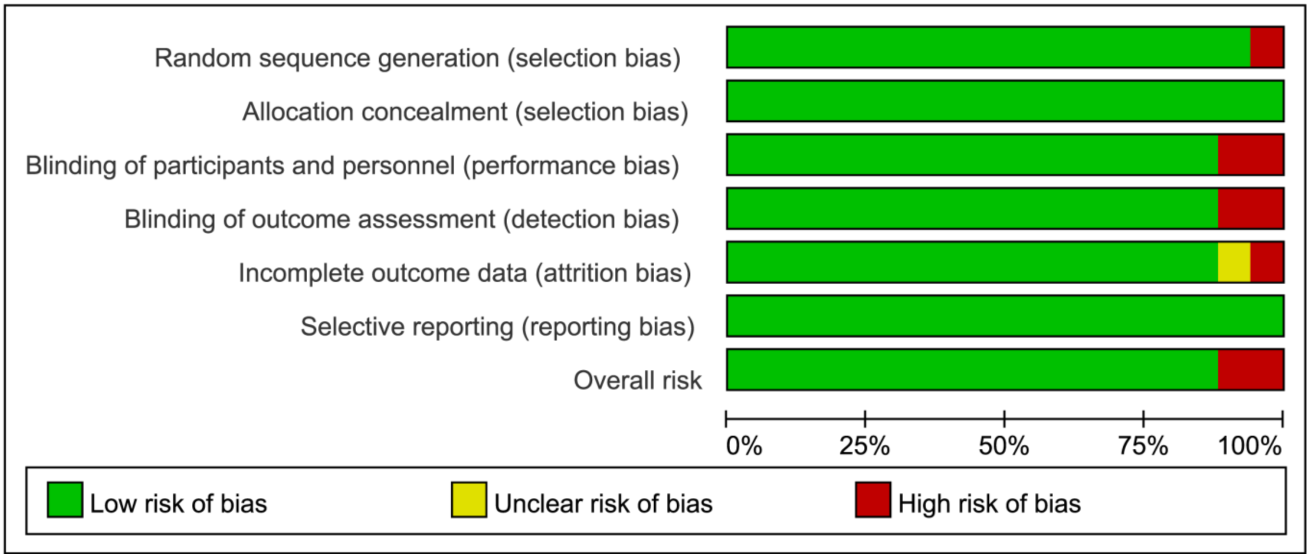

Supplementary Figure 9. the Risk of bias graph With RevMan 5.3

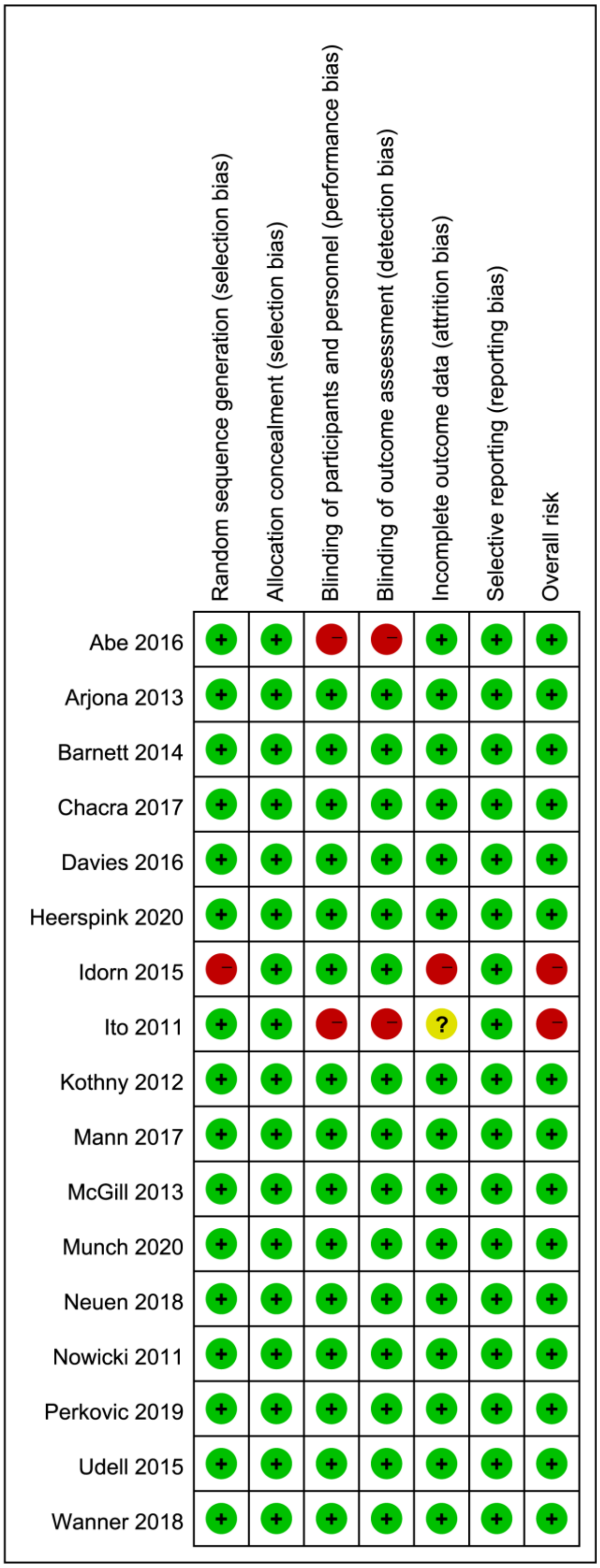

Supplementary Figure 10. the Risk of Bias summary table With RevMan 5.3

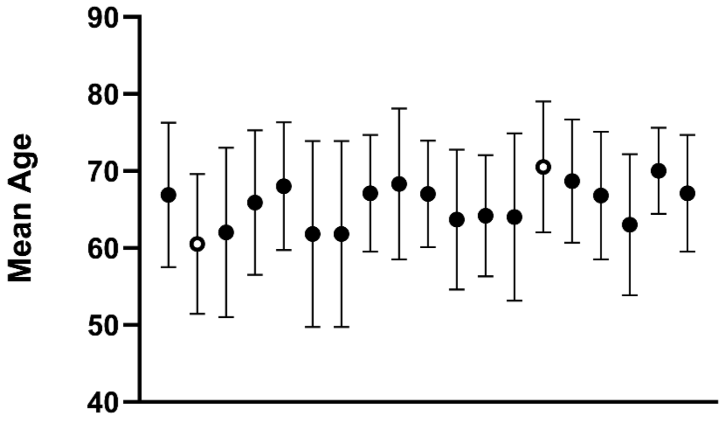

Supplementary Figure 11. Mean age distribution of all included studies

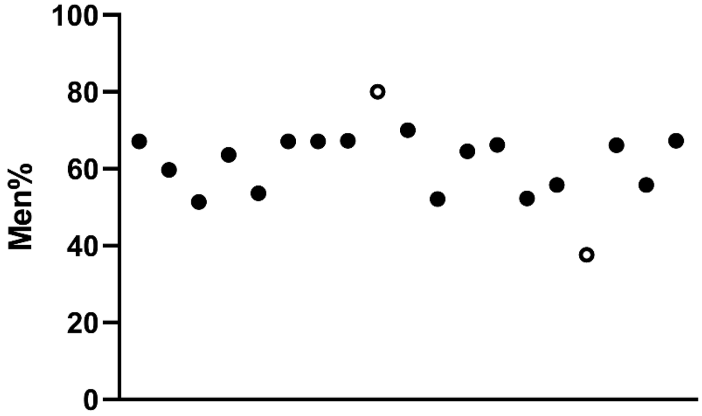

Supplementary Figure 12. Men proportion of all included studies

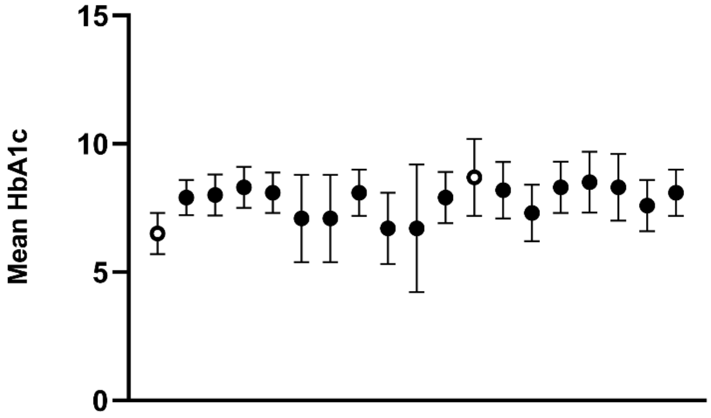

Supplementary Figure 13. Mean HbA1c distribution of all included studies

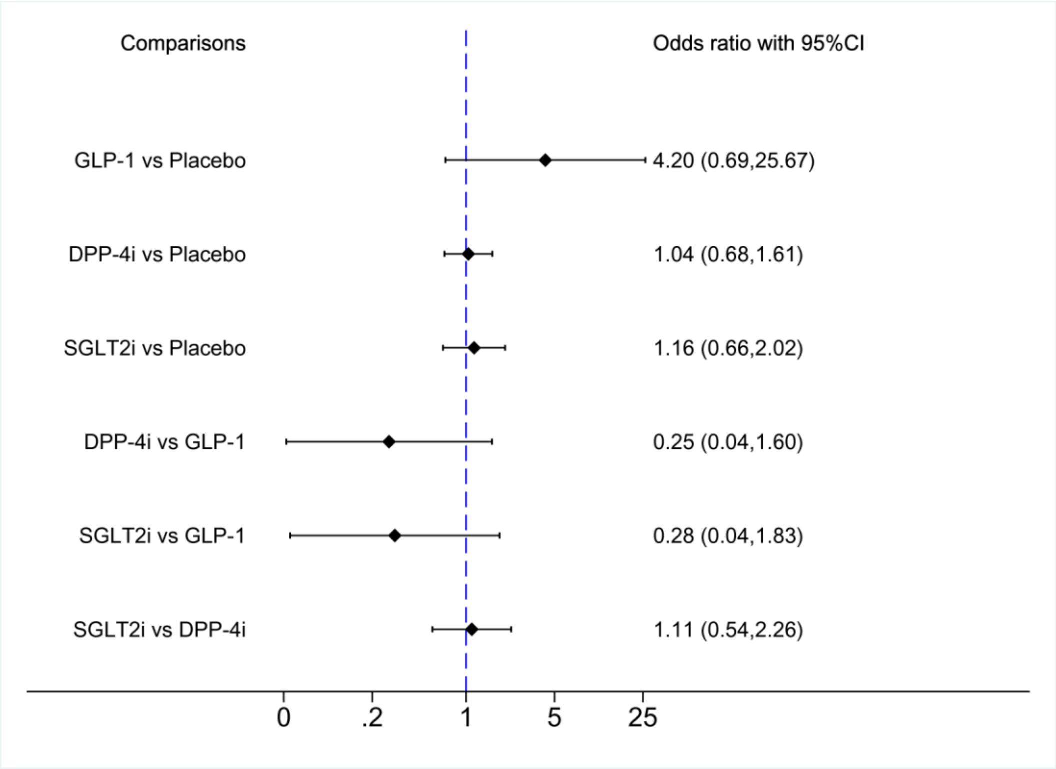

Supplementary Figure 14. Forest plot of odds ratio comparing the hypoglycemia events between each medicine class or between each medicine class and placebo

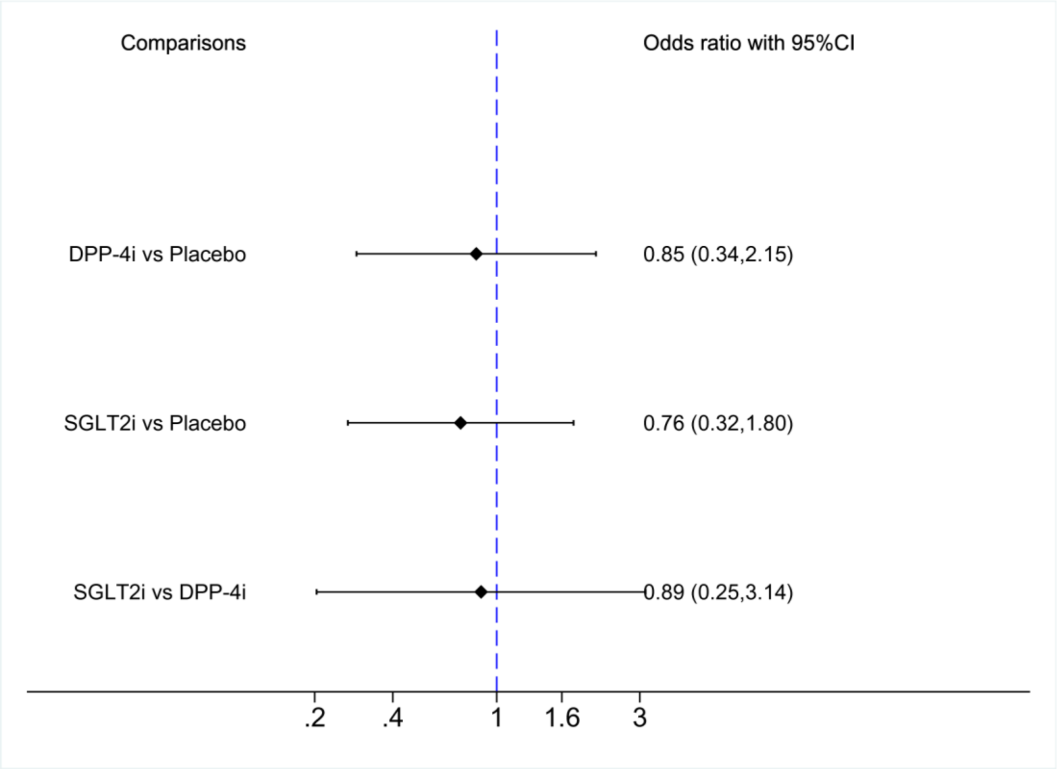

Supplementary Figure 15. Forest plot of odds ratio comparing the severe hypoglycemia events between each medicine class or between each medicine class and placebo

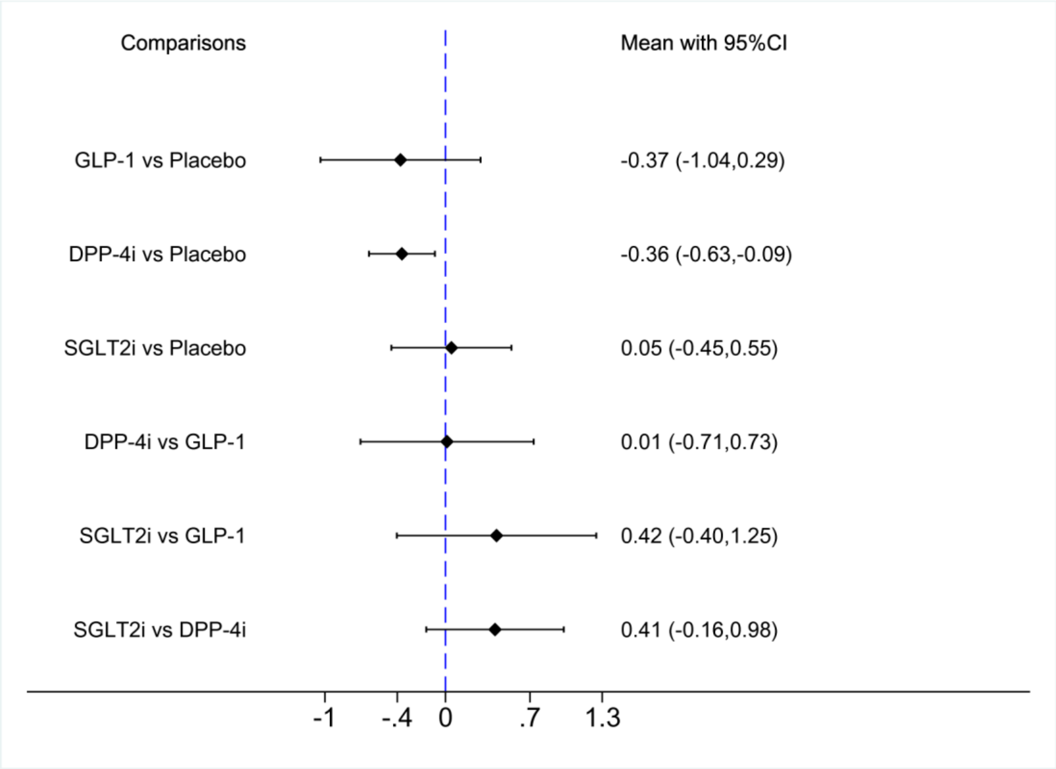

Supplementary Figure 16. Forest plot of mean difference comparing the change from baseline in HbA1c between each medicine class or between each medicine class and placebo

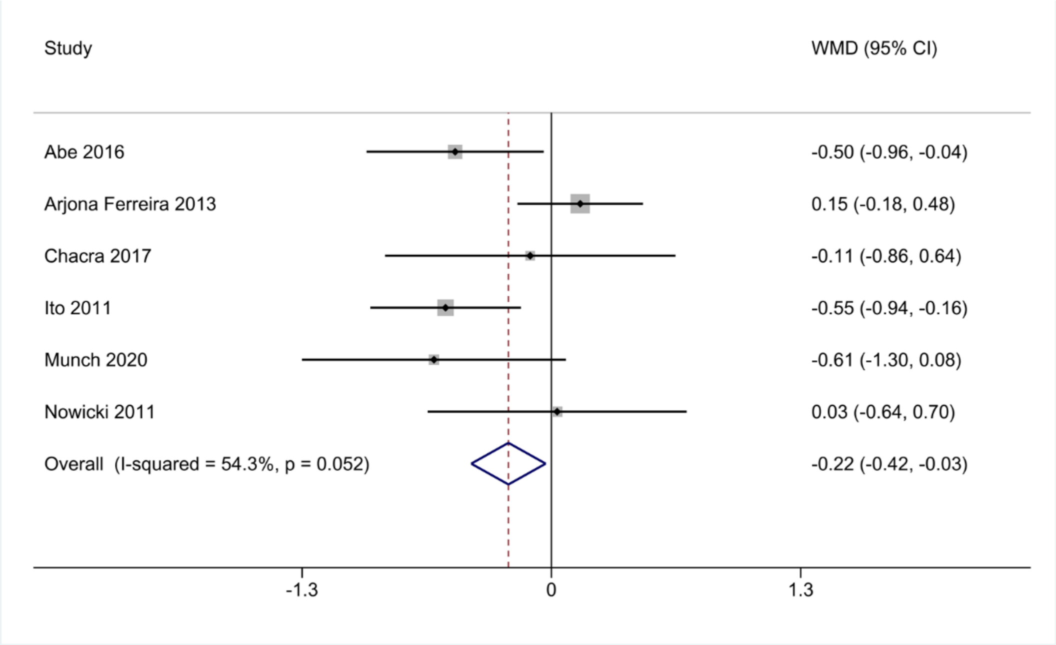

Supplementary Figure 17. Forest plot of mean difference comparing the change from baseline in HbA1c between DPP-4i and placebo among patient with type 2 diabetes on hemodialysis

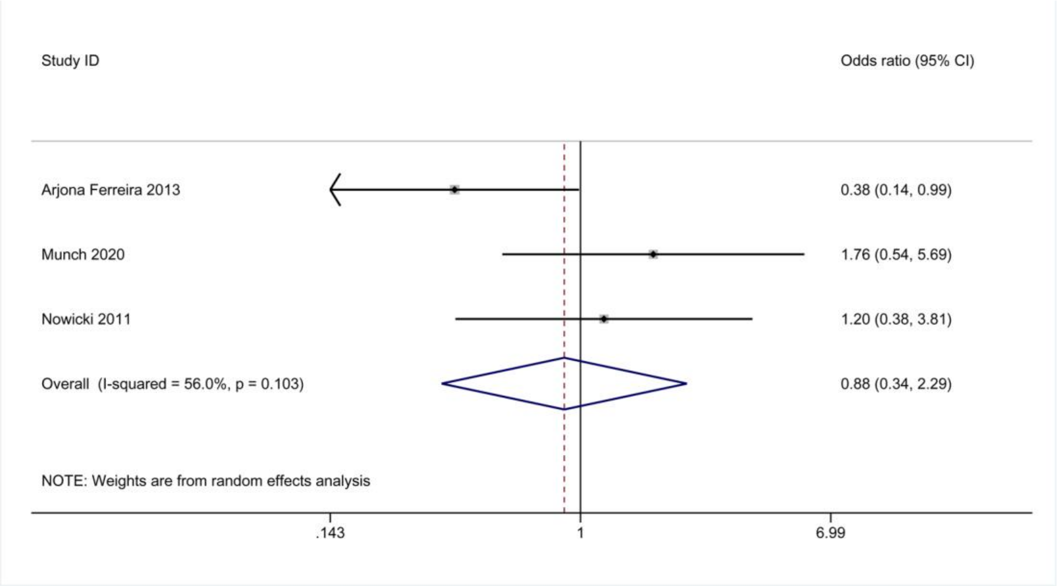

Supplementary Figure 18. Forest plot of odds ratio comparing the hypoglycemia events between DPP-4i and placebo among patient with type 2 diabetes on hemodialysis

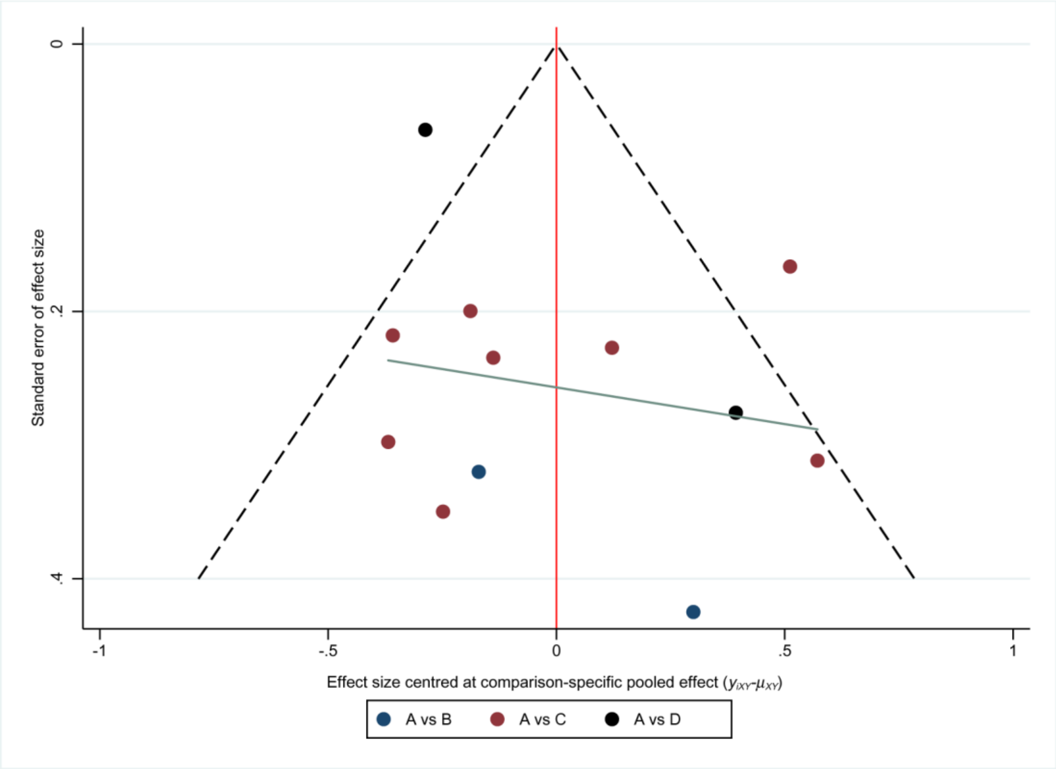

Supplementary Figure 19. Funnel plot of mean difference comparing the change from baseline in HbA1c between each medicine class or between each medicine class and placebo

2.2 Supplementary Tables

Supplementary Table 1 Pairwise meta–analysis

Comparison	Trail	OR	95% CI
**Serious AE**			
GLP-1RA vs PLA	Davies 2016	0.962	0.353, 2.619
	Idorn 2015	6.75	0.662, 68.779
	**Pooled OR**	**1.908**	**0.304,11.966**
	**Heterogeneity (I²)**	***56.80%***	
DPP-4i vs PLA	Arjona Ferreira 2013	1.175	0.567, 2.436
	Kothny 2012	0.972	0.466, 2.028
	McGill 2013	0.818	0.407, 1.643
	**Pooled OR**	**0.973**	**0.642, 1.474**
	**Heterogeneity (I²)**	**0.00%**	
SGLT2i vs PLA	Barnett 2014	1.142	0.415, 3.141
	Neuen 2018	0.614	0.435, 0.866
	**Pooled OR**	**0.693**	**0.427, 1.122**
	**Heterogeneity (I²)**	**23.00%**	
**Serious renal-related adverse events or renal death**			
DPP-4i vs PLA	McGill 2013	1.21	0.310, 4.721
	Udell 2015	1.076	0.684, 1.692
	**Pooled OR**	**1.089**	**0.709, 1.673**
	**Heterogeneity (I²)**	**0.00%**	
SGLT2i vs PLA	Herrington 2018	0.802	0.503, 1.278
	Neuen 2018	0.886	0.337, 2.330
	Perkovic2019	0.699	0.516, 0.947
	Heerspink 2020	0.646	0.516, 0.808
	Pooled OR	***0.686***	***0.581, 0.810***
	**Heterogeneity (I²)**	**0.00%**	
**All-cause mortality**			
DPP-4i vs PLA	Arjona Ferreira 2013	0.656	0.176, 2.442
	Kothny 2012	2.077	0.211, 20.425
	McGill 2013	0.954	0.185, 4.906
	Munch 2020	0.333	0.013, 8.489
	**Pooled OR**	**0.833**	**0.339, 2.046**
	**Heterogeneity (I²)**	**0.00%**	
SGLT2i vs PLA	Barnett 2014	0.189	0.009, 4.082
	Wanner 2018	0.844	0.507, 1.405
	Heerspink 2021	0.684	0.495, 0.945
	Pooled OR	***0.719***	***0.548, 0.944***
	**Heterogeneity (I²)**	**0.00%**	
**Hypoglycemia**			
DPP-4i vs PLA	Arjona Ferreira 2013	0.376	0.143, 0.989
	Kothny 2012	1.064	0.461, 2.452
	McGill 2013	1.774	0.888, 3.545
	Munch 2020	1.761	0.545, 5.692
	Nowicki 201	1.2	0.378, 3.806
	Udell 2015	0.747	0.298, 1.876
	**Pooled OR**	**1.033**	**0.642, 1.662**
	**Heterogeneity (I²)**	**36.40%**	

Supplementary Table 1 (*continued.*)

Comparison	Trail	OR	95% CI
**Serious AE**			
SGLT2i vs PLA	Barnett 2014	1.268	0.487, 3.301
	Wanner 2018	1.116	0.734, 1.697
	**Pooled OR**	**1.14**	**0.776, 1.673**
	**Heterogeneity (I^2)**	**0.00%**	
**Severe hypoglycemia**			
DPP-4i vs PLA	McGill 2013	0.954	0.185, 4.906
	Munch 2020	1.033	0.137, 7.815
	Udell 2015	0.731	0.193, 2.773
	**Pooled OR**	**0.854**	**0.340, 2.145**
	**Heterogeneity (I^2)**	**0.00%**	
SGLT2i vs PLA	Barnett 2014	1	0.060, 16.611
	Herrington 2018	0.736	0.296, 1.832
	**Pooled OR**	**0.758**	**0.318, 1.804**
	**Heterogeneity (I^2)**	**0.00%**	
**Change of HbA1c**			
Comparison	Trail	OR	95% CI
GLP-1RA vs PLA	Davies 2016	-0.57	-0.929, -0.211
	Idorn 2015	-0.1	-0.933, 0.733
	**Pooled *WMD***	***-0.491***	***-0.835, -0.147***
	**Heterogeneity (I^2)**	**3.10%**	
DPP-4i vs PLA	Abe 2016	-0.5	-0.960, -0.040
	Arjona Ferreira 2013	0.15	-0.176, 0.476
	Chacra 2017	0.21	-0.401, 0.821
	Ito 2011	-0.55	-0.941, -0.159
	Kothny 2012	-0.73	-1.313, -0.147
	McGill 2013	-0.72	-1.147, -0.293
	Munch 2020	-0.61	-1.296, 0.076
	Nowicki 2011	-0.24	-0.685, 0.205
	**Pooled *WMD***	***-0.362***	***-0.634, -0.089***
	**Heterogeneity (I^2)**	***62.90%***	
SGLT2i vs PLA	Barnett 2014	0.48	-0.061, 1.021
	Wanner 2018	-0.2	-0.326, -0.074
	**Pooled WMD**	**0.087**	**-0.571, 0.745**
	**Heterogeneity (I^2)**	***82.70%***	

Note: Pooled effect size in black italics indicates statistical significance, and Heterogeneity (I^2) in black italics indicates moderate or above heterogeneity

AE: Adverse event; DPP-4i: dipeptidyl peptidase-4 inhibitors; GLP-1RA: glucagon-like peptidyl-1 receptor agonists; OR: Odds ratio; PLA: Placebo; SGLT2i: Sodium-glucose cotransporter 2 inhibitors; WMD: weighted mean difference
